# Options for affordable pre-exposure prophylaxis (PrEP) in national HIV prevention programmes in Europe

**DOI:** 10.2807/1560-7917.ES.2017.22.42.17-00698

**Published:** 2017-10-19

**Authors:** Rosalind Coleman, Maria Prins

**Affiliations:** 1 PrEP consultant, The Joint United Nations Programme on HIV/AIDS (UNAIDS), Geneva, Switzerland; 2 Public Health Service of Amsterdam and Academic Medical Center, Amsterdam, The Netherlands

**Keywords:** pre-exposure prophylaxis, HIV prevention, cost-effectiveness, budget impact, access to medicine

The successful integration of pre-exposure prophylaxis (PrEP) into comprehensive HIV programmes that include increased testing, the offer of early treatment for infected individuals and combination HIV prevention, is showing signs of contributing to a reduction in new HIV infections [1,2]. Such programmes can enable and motivate people with a high risk of HIV infection to come for testing, encourage those who test negative to consider PrEP, support their effective adherence and deliver collateral benefits such as increased screening and treatment of other sexually transmitted infections (STIs) [1,3]. This promising evidence, in the context of stagnant or rising incidence of HIV in many European countries [4], has naturally led to intensifying demand for inexpensive and broader provision of PrEP [5].

The great majority of current PrEP users in Europe and other similar settings are gay and bisexual men and other men who have sex with men (MSM) at high risk of HIV-infection [3,5,6]. They are generally well-informed, motivated and supported by civil society and concerned clinicians. However, even in well-established PrEP programmes that engage with less empowered populations, building up to more extensive distribution and uptake of PrEP remains a challenge [7]. All PrEP programmes need to address persistent barriers and doubts including the need for an estimate of the number of people eligible for PrEP, the price of the PrEP medication, the risk of drug resistance, a potential increase in STI diagnoses via risk compensation and increased testing, achieving effective adherence to PrEP and limited engagement with PrEP by certain members of key populations and certain healthcare providers [6]. Despite some encouraging experience around meeting these concerns [1,2,6] the persistent uncertainty weakens estimates of cost-effectiveness for PrEP and hinders planning for broader implementation. Countries are faced with the dilemma of how to implement and fund effective PrEP programmes at a national scale in a way that addresses need, minimises possible negative impact and remains within the country’s means [5,6]. Secure integration with other sexual health and community services has the potential to bring out the collateral benefits of PrEP access [5].

The economic evaluation of PrEP in England by Ong et al., published in this edition of *Eurosurveillance* [8], explores implications of the first phase of a PrEP programme for MSM at high HIV risk. Despite its limitations, the static decision analytic model that was chosen is attractive due to its simplicity that encourages a broader engagement with cost-effectiveness analyses. The model’s short time relevance reflects the difficulties of projecting PrEP costs and effects very far into the future. Limitations also include that effects beyond the benefit to individuals receiving PrEP could not be modelled using this approach, so the total benefits of PrEP might be underestimated.

The results emphasise the high sensitivity of PrEP cost effectiveness to (i) the price of the medicine, (ii) the HIV risk of those taking PrEP and (iii) their level of adherence. This is in line with findings from other modelling studies from high income countries, using various approaches, that indicate that PrEP programmes will become more cost-effective or even cost-saving if PrEP is used by groups (of MSM) who are at the highest risk of HIV infection and when medication costs are reduced, including potential savings through the uptake of on-demand PrEP [5,6,9-13].

PrEP is evaluated as potentially cost effective in England if taken up with good adherence and correspondingly high clinical effectiveness by groups with a ca 3 per 100 person years’ risk of HIV infection [8]. The uncertainty around these parameters, and the sensitivity of cost-effectiveness estimates to them, did not allow for stronger conclusions to be drawn.

The budgetary impact of a modest programme was considerable: in a single year, a PrEP service for 5,000 PrEP person years costs €36.6M (£26.9M) at current British National Formulary (BNF) price of the patented drug. Since the price of the PrEP medicine is the main budgetary cost, it is crucial that ways be found to reduce this if PrEP programmes are to go to scale. Different funding models for PrEP have been explored, depending on country health programme frameworks, but the price of the PrEP medicine limits how many people will be offered it whether funding is central, through insurance programmes or private [14].

We take this opportunity to review various strategies to access affordable antiretrovirals for PrEP.

In France, a national programme with subsidised costs has been rolled out since January 2016 [5]. Norway [15] and Scotland [16] have indicated that PrEP will be free to the person using it, and other European countries are taking first steps to roll out PrEP [17]. Otherwise, a number of potential PrEP users find that PrEP programmes have been too slow to come to scale to meet their needs [18]. Different routes to obtain PrEP, have thus been opened through civil society [18] to obtain generic PrEP, mainly via the Internet. Where there is pro-active clinical support, the safety and follow-up of people using PrEP purchased online can be assured. There are, however, Internet sites that require neither a prescription, nor any other proof of a negative HIV test or renal function result in order to pass on the order for PrEP.

Until the end of 2016, the only medicine to be approved and marketed as PrEP in high income countries was the patented version of the fixed-dose oral combination of tenofovir disoproxil fumarate with emtricitabine (TDF/FTC). Beyond negotiating with patent holders, the way for countries to access antiretrovirals for national PrEP programmes at affordable prices may be via market competition between multiple manufacturers, i.e. including generic manufacturers. It is of particular relevance to European PrEP that several generic manufacturers have recently received marketing approval from the European Medicines Agency for tenofovir disoproxil with emtricitabine (TDX/FTC) that is bioequivalent to TDF/FTC [19]. This can now be marketed in countries where it does not infringe a patent. Several generic manufacturers are already supplying their TDX/FTC as PrEP [20]. There are also provisions within the World Trade Organization’s *Agreement on Trade-Related Aspects of Intellectual Property Rights* (TRIPS) that countries may apply to access generically manufactured medicines, depending on the necessary national laws being in place [21,22]. Australia, for example, was the first country to use a TRIPS provision to access PrEP at a meaningful scale. The provision allows research with patented medicines in order to understand the medicine more fully [21-23].

The PROUD trial in England [24], showed in 2014 that daily PrEP, introduced through existing sexual health clinics in addition to standard-of-care risk reduction, reduced the HIV incidence by 86%. Questions remained about estimation of PrEP need, cost-effectiveness and the budgetary impact of the available branded drug, so the PrEP Impact trial was proposed to prepare the way for full roll-out [25]. The trial, that started recruiting in October 2017, will involve 10,000 participants in a pragmatic health technology assessment of PrEP implementation, investigating eligibility, uptake and duration of use, as well as impact on HIV and other STIs. The English trial, similarly to the one undertaken in Australia, uses generic TDF/FTC under national legislation [26]. The results will be used to support future clinical and cost-effective PrEP access [25].

## Examples of generic PrEP prices in Europe

In England, a generic drug manufacturer won the Impact trial drug supply contract through a competitive tendering process [27], but, the eventual price agreed remains confidential. Given that national and local research costs and the trial drug for 20,000 person years of PrEP use is being paid for out of a total £10 million budget [25], it may be deduced that the price of the trial drug should be considerably cheaper than the current BNF price of the patented drug.

In the Netherlands, different formulations of TDX/FTC are available costing ca 75% of the current Dutch price of the patented drug [28,29]. Although both the patented and generic formulations are currently reimbursed for the treatment of HIV, the level of reimbursement when used as PrEP remains to be determined [30]. The city council of Amsterdam has already committed to pay for healthcare for PrEP users who buy PrEP abroad or obtain it through other means [31]. Meanwhile, the Dutch Health Council is formulating guidance on PrEP use to present to the Minister for Health, Welfare and Sport who decides on PrEP implementation and will be in charge of negotiating PrEP access for a national programme at an affordable price [30].

In parallel to the budgetary impact for governments, the out of pocket costs to the individual users will determine the uptake of PrEP. Internet purchasing prices in Europe are quoted as around €45 (40£) per month which is similar to the recent out-of-pocket cost that has been negotiated in Germany’s national PrEP roll-out [32,33].

Internet purchasing, however, is neither a viable or safe long-term substitute for national PrEP programmes, nor legal in all countries. The growth of buyers’ clubs demonstrates the demand for PrEP and can act as a stimulus to national programme planners to explore ways of purchasing PrEP medicine and making it available in affordable and safely regulated programmes.

In conclusion, as demonstrated by the paper of Ong and Gill [8], for a PrEP programme to be sustainable and cost-effective, affordable PrEP needs to be chosen and used appropriately by people at substantial risk of HIV infection. Secure integration with supportive sexual health and community services will have the greatest possibility to bring out the collateral benefits of PrEP access. Since the biggest component of the initial budget impact is the cost of the medicine, active steps are required to enable access to medicine at affordable costs. The European Medicines Agency approval of TDX/FTC represents an opportunity to further advance the roll-out of PrEP although price negotiations and intellectual property legislation review are required on a country-by-country basis.
